# Postoperative Pain and Opioid Use Following Lower-Limb Escharectomy and Skin Grafting Under a Standardized Regional Anesthesia Protocol: A Retrospective Study

**DOI:** 10.3390/life16020202

**Published:** 2026-01-26

**Authors:** Francesco Coppolino, Francesco Coletta, Antonio Tomasello, Pasquale Rinaldi, Maria Rosaria Cavezza, Romolo Villani, Francesca Schettino, Ilaria Mataro, Antonio Scalvenzi, Caterina Aurilio, Pasquale Sansone, Maria Caterina Pace, Vincenzo Pota

**Affiliations:** 1 Department of Women, Child, General and Specialistic Surgery, University of Campania “L. Vanvitelli”, 80138 Naples, Italy; francesco.coppolino@unicampania.it (F.C.); mariacaterina.pace@unicampania.it (M.C.P.); caterina.aurilio@unicampania.it (C.A.); 2 Emergency Anaesthesia, Burn Intensive Care Unit and Poison Control, AORN Antonio Cardarelli Hospital, 80131 Naples, Italy; dottfrancescocoletta@gmail.com (F.C.); pasquale.rinaldi1994@gmail.com (P.R.); romolo.villani@aocardarelli.it (R.V.);; 3 Plastic and Reconstructive Surgery and Burn Unit, AORN Antonio Cardarelli Hospital, 80131 Naples, Italy

**Keywords:** burn patients, regional anesthesia, postoperative pain management, opioid dependence, escharectomy, skin graft

## Abstract

Background: Pain management in patients with severe burns remains one of the most complex challenges in perioperative care. Burn-related pain is multifactorial, resulting from tissue destruction, intense inflammation, surgical procedures, and repeated dressing changes. Opioids remain the cornerstone of analgesia; however, prolonged use is associated with tolerance, dependence, adverse effects, and prolonged hospitalization. Multimodal and opioid-sparing strategies, including regional anesthesia, may improve postoperative outcomes by enhancing analgesia while reducing systemic drug exposure. This study aimed to evaluate the effectiveness of a standardized regional anesthesia protocol in reducing postoperative pain and opioid requirements in burn patients undergoing lower-limb escharectomy and autologous skin grafting. Methods: We conducted a retrospective, single-center analysis of 25 adult patients with deep thermal burns of the lower limbs who underwent escharectomy and split-thickness skin grafting. All patients received a combined ultrasound-guided sciatic popliteal block and adductor canal block on both the burned limb and the donor site. Ropivacaine 0.375% with clonidine was administered without exceeding a total dose of 3.0 mg/kg. Postoperative pain was assessed using the Numerical Rating Scale (NRS), and opioid consumption was recorded as rescue doses in intravenous morphine equivalents. Secondary outcomes included perioperative complications and 30-day hospital readmission. Results: Regional anesthesia provided effective postoperative pain control. Thirty-two percent of patients reported no pain (NRS 0), 52% reported mild pain (NRS 1–3), and 16% reported moderate pain (NRS 4–6). No patient reported severe pain (NRS 7–10). Only four patients (16%) required rescue opioids. No perioperative complications or block-related adverse events occurred, and no patient required hospital readmission within 30 days. Conclusions: In this cohort, regional anesthesia was associated with satisfactory postoperative analgesia and minimal opioid requirements. By reducing opioid exposure, this approach may help improve patient comfort and potentially limit opioid-related adverse effects. Larger prospective studies are needed to confirm these findings and to assess long-term outcomes.

## 1. Introduction

Pain management in patients with severe burns represents a major clinical challenge. Burn injuries require a multidisciplinary approach involving plastic and reconstructive surgeons, intensive care specialists, anesthesiologists, rehabilitation teams, and specialized nursing staff, given the complex clinical and psychological needs of these patients [1,2]. Burn injuries may result from thermal, chemical, or electrical mechanisms and frequently affect elderly or medically fragile individuals [3]. Despite substantial advancements in perioperative burn care, pain control remains highly variable and often inadequate across different stages of treatment. Despite the growing literature on pain management in burn patients, procedure-specific evidence focusing on lower-limb escharectomy remains limited. Lower-limb escharectomy combines extensive wound surfaces with the additional pain generated by skin graft harvesting, resulting in a postoperative pain burden that differs from that of other lower-limb surgical procedures more commonly reported in the literature [4].

Opioids continue to play a central role in analgesic and sedative regimens for most patients admitted to the intensive care unit (ICU) [5]. However, insufficient analgesia may contribute to the development of chronic pain syndromes, including Chronic Post-ICU Pain (CPIP) [6], whereas prolonged or high-dose opioid exposure carries risks of tolerance, dependence, and immuno-endocrine dysregulation [7]. For these reasons, contemporary critical care increasingly aims to optimize acute pain management while reducing unnecessary long-term opioid exposure.

Extensive evidence—including randomized trials, donor-site analgesia protocols, meta-analyses, and expert guideline recommendations—has demonstrated that regional anesthesia and peripheral nerve blocks can reduce postoperative pain, improve functional outcomes, and decrease systemic opioid requirements in burn patients. Nevertheless, the implementation of regional techniques remains heterogeneous, and standardized protocols tailored to specific burn procedures are still limited.

In this context, the present retrospective study aims to provide an incremental contribution by evaluating a structured and reproducible protocol combining bilateral sciatic popliteal block with adductor canal block for adult burn patients undergoing lower-limb escharectomy and autologous skin grafting. The study aims to assess postoperative pain trajectories and opioid requirements within this standardized approach, offering real-world data that may support broader protocolization in similar clinical settings.

## 2. Patients and Methods

This study was conducted in accordance with the ethical principles of the Declaration of Helsinki and was approved by the Comitato Etico Campania 3 (COM ETICO CAMP3, protocol number 23/05/2025). A retrospective, single-center analysis was performed in the Burn Intensive Care Unit of AORN Antonio Cardarelli Hospital, Naples, Italy. The study included 25 adult patients admitted between January 2024 and March 2025 who required lower-limb escharectomy followed by autologous split-thickness skin grafting.

All enrolled patients had lesions—although not of surgical or segmental relevance—also involving the upper part of the leg, and this site was considered suboptimal for harvesting. In our burn unit, escharectomy followed by autologous skin grafting is routinely performed between day 3 and day 5 after burn injury, according to the patient’s clinical stability and wound conditions. In light of the presence of partial lesions and the possibility of harvesting from a cosmetically unfavorable area, the contralateral limb was selected in order to optimize both functional and aesthetic outcomes.

**Inclusion criteria were:**

• Age ≥18 years;
• Deep dermal or full-thickness burns of the lower limbs;
• Planned escharectomy and skin grafting under regional anesthesia;
• Ability to undergo postoperative pain assessment using the Numerical Rating Scale (NRS).

**Exclusion criteria were:**

• Pre-existing neurological deficits of the lower limbs;
• Allergy to local anesthetics or clonidine;
• Chronic opioid therapy prior to hospitalization;
• Psychiatric or cognitive conditions impairing pain assessment.

All procedures were performed in a dedicated sterile environment by anesthesiologists experienced in ultrasound-guided regional anesthesia. Standard monitoring included continuous electrocardiography, non-invasive blood pressure, pulse oximetry (SpO_2_), and respiratory rate. Mild sedation with midazolam (0.05 mg/kg) was administered for patient comfort.

For the limb undergoing escharectomy, an ultrasound-guided sciatic popliteal nerve block was performed using a high-frequency linear probe (8–12 MHz) with an in-plane technique. A total of 20 mL of ropivacaine 0.375% combined with clonidine (0.5 µg/kg) was injected around the sciatic nerve at the popliteal level. To ensure complete perioperative analgesia of the anterior leg, an adductor canal block was subsequently administered using 10 mL of ropivacaine 0.375% with clonidine (0.2 µg/kg).

The same protocol was applied to the contralateral, unburned limb from which the autologous skin graft was harvested, ensuring symmetrical sensory coverage of both operative sites. In all cases, the cumulative dose of ropivacaine did not exceed 3.0 mg/kg. Sensory block efficacy was confirmed using cold testing and pinprick assessment prior to incision.

All blocks were performed in accordance with international safety guidelines regarding anticoagulation, and no patient received low-molecular-weight heparin within the time window contraindicated for peripheral nerve blocks. No continuous catheter technique was used. Sedation with low-dose midazolam or dexmedetomidine was allowed at the anesthesiologist’s discretion, but no patient required conversion to general anesthesia. Hemodynamic parameters were recorded at 5 min intervals throughout the procedure.

After surgery, all patients were monitored in the high-dependency burn recovery unit. Pain intensity was assessed at rest and during mobilization using the 11-point Numerical Rating Scale (NRS: 0 = no pain, 10 = worst imaginable pain) during the first 24 h.

Postoperative rescue analgesia was standardized: intravenous paracetamol (1 g every 8 h) was administered routinely, while opioids (expressed as intravenous morphine equivalents) were administered only if the NRS score was ≥4. Any nerve block–related adverse events (hematoma, infection, local anesthetic systemic toxicity, persistent neurological deficits) were recorded. Hospital readmissions within 30 days of discharge were retrieved from electronic medical records.

Pain during wound care and dressing changes was also assessed. Wound care and the first dressing change were routinely performed 5 days after escharectomy and autologous split-thickness skin grafting. Subsequent dressing changes were performed on alternate days until complete wound healing was achieved. Pain was assessed using the NRS, with mean scores ranging from 0 to 2, as the wounds were covered by a well-adhered skin graft and did not generate clinically relevant pain. Similarly, pain at the donor sites never exceeded an NRS score of 2, as only the external dressings were changed, allowing spontaneous re-epithelialization without traumatic manipulation.

**Primary endpoints were:**

• Postoperative pain intensity (NRS);
• Need for rescue opioid administration (intravenous morphine equivalents).

**Secondary endpoints included:**

• Block-related complications;
• Length of postoperative stay;
• Hospital readmission within 30 days.

Data were analyzed using descriptive statistics. Continuous variables were expressed as mean ± standard deviation or median with interquartile range, as appropriate. Categorical variables were presented as absolute numbers and percentages. Given the limited sample size (*n* = 25), inferential statistical testing was not performed, as the aim was to describe outcomes within a homogeneous cohort treated using an identical protocol. Descriptive analysis was therefore considered appropriate for this retrospective clinical evaluation.

## 3. Results

A total of 25 adult patients undergoing lower-limb escharectomy followed by autologous split-thickness skin grafting were included in the analysis (Table 1). All patients received the same standardized regional anesthesia protocol consisting of ultrasound-guided sciatic popliteal and adductor canal nerve blocks. No patient required conversion to general anesthesia or additional intraoperative analgesic interventions. **Baseline characteristics** of the study population are summarized in Table 1. The mean age was 54 ± 12 years (range 29–73), with a predominance of male patients (60%). The mean total body surface area (TBSA) involved was 12 ± 4%, with deep dermal burns in 48% of cases and full-thickness burns in 52%. Circumferential involvement was present in 36% of patients. The most frequently involved anatomical regions were the lower leg (52%), thigh (28%), and foot (20%). The most common comorbidities were hypertension (32%) and diabetes mellitus (24%).

Postoperative pain control was consistently satisfactory across the cohort. Pain intensity was assessed using the Numerical Rating Scale (NRS) at rest and during mobilization according to standard nursing documentation. Overall, 8 patients (32%) reported complete absence of pain (NRS 0), 13 patients (52%) experienced mild pain (NRS 1–3), and 4 patients (16%) reported moderate pain (NRS 4–6). Importantly, no patient experienced severe pain (NRS 7–10) at any postoperative time point. Specifically, all patients received intravenous paracetamol at a fixed dose of 1 g every 8 h. Intravenous morphine was used exclusively as rescue therapy, at a dose ranging from 0.03 to 0.05 mg/kg, in cases of inadequate pain control.

**Figure 1:**
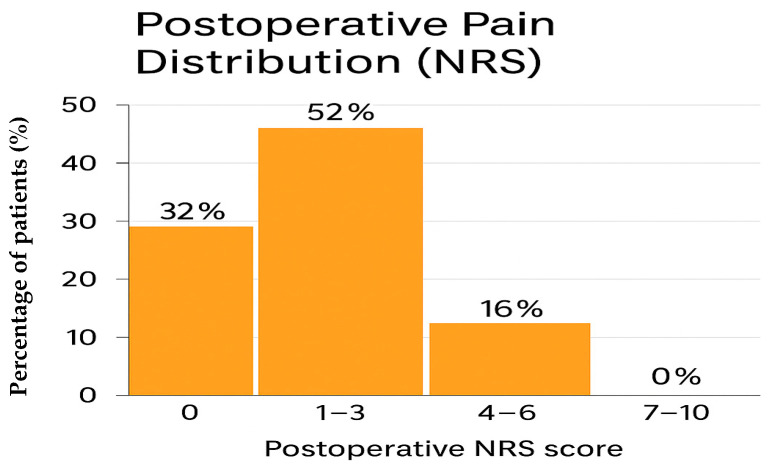
Distribution of postoperative pain scores (NRS) in the study population.

Only 4 of 25 patients (16%) required at least one dose of rescue morphine during the postoperative period (Table 2). The distribution of pain scores is presented in Table 2 and summarized in Figure 1.

In all patients reporting NRS 0 or mild pain, analgesia was considered clinically adequate and no additional interventions were required.

No episodes of opioid-related respiratory depression, nausea or vomiting requiring treatment, or pruritus were documented.

The remaining 21 patients (84%) achieved adequate pain control with regional anesthesia supplemented exclusively by scheduled intravenous paracetamol, confirming a substantial reduction in opioid exposure in the early postoperative period.

**Safety outcomes.** No perioperative complications related to the nerve blocks were recorded. Specifically, there were:

• no vascular punctures,
• no episodes of local anesthetic systemic toxicity (LAST),
• no neurological deficits at 24 or 48 h,
• no hematoma or infection at the needle insertion site.

Hemodynamic stability was maintained throughout both the intraoperative and immediate postoperative periods. No block failures occurred, and no patient required conversion to general anesthesia.

All patients were discharged according to standard postoperative burn unit protocols. No readmissions within 30 days of discharge were observed (Table 3). Notably, no patient returned because of uncontrolled pain or opioid-related adverse effects.

## 4. Discussion

The management of burn pain remains a significant clinical challenge due to the complex interaction of anatomical, physiological, pharmacological, psychosocial, and pre-existing patient factors [8]. Burn pain is particularly severe and persistent, involving background, procedural, and postoperative components. Its pathophysiology includes direct nerve injury, intense inflammatory responses, and central sensitization, which may progress to chronic pain syndromes if inadequately controlled [8,9,10]. Without effective and timely analgesia, patients are at risk of both acute suffering and long-term sequelae, including anxiety, post-traumatic stress disorder, impaired wound healing, and delayed rehabilitation. Pain management in burn patients is further complicated by the absence of universally accepted protocols, with clinical practice varying widely across institutions.

Severe burns cause extensive tissue destruction and trigger a substantial cytokine-mediated inflammatory response that may contribute to both local and systemic complications [11]. The increase in inflammatory mediators in burn patients remains an active area of investigation, with important implications for wound repair and systemic inflammation [12]. The systemic consequences of major burns occur in two distinct phases: an initial burn-shock phase, characterized by hypovolemia and circulatory failure, followed by a hypermetabolic phase first described by Cuthbertson in 1942 [13]. Understanding these physiological alterations is essential for clinicians involved in perioperative and critical care management. Patients with extensive burns often develop marked edema, including in uninjured tissues, due to continuous plasma leakage during the first 48 h [14]. Unlike other forms of trauma, burn-related fluid loss does not immediately reduce red blood cell mass; instead, hemoconcentration is typical.

Early resuscitation therefore focuses on restoring intravascular volume to optimize perfusion and limit ischemic injury. Burn shock, resulting from reduced circulating volume and increased systemic vascular resistance mediated by catecholamine and vasopressin release, may persist for up to 36 h. As patients transition to the hypermetabolic phase, they develop increased oxygen consumption, carbon dioxide production, cardiac output, tachycardia, and profound protein catabolism. Burn-related inflammatory pain, compounded by surgical interventions, remains a major barrier to recovery [4]. Consequently, opioid therapy continues to represent the cornerstone of analgesia for severe burns [15]. However, prolonged opioid exposure increases the risk of tolerance and dependence and contributes to post-discharge readmissions and higher healthcare costs [16].

Intravenous opioids allow rapid titration and predictable analgesia, but dosing must be individualized because of altered pharmacokinetics in burn patients and the risk of opioid tolerance or opioid-induced hyperalgesia [6,17,18,19,20,21]. Tolerance requires progressively higher doses to achieve the same analgesic effect, whereas dependence is a chronic neurobiological condition characterized by compulsive drug use and psychological craving. Evidence regarding the prevalence of chronic opioid use after burn injury in ICU settings remains limited. Clinicians must therefore balance the risk of undertreating acute pain—potentially leading to chronic pain—with the dangers of excessive opioid exposure and long-term dependence. Both inadequate analgesia and chronic opioid use can have substantial physical, psychological, and socioeconomic consequences for patients and caregivers [22]. Structured post-ICU follow-up is essential for identifying persistent pain and tailoring long-term analgesic strategies.

Modern critical care emphasizes multimodal analgesia in order to reduce opioid requirements while maintaining effective pain control. The American Burn Association and other expert panels strongly recommend integrating non-opioid adjuncts with opioids to limit cumulative opioid dose [19]. Although no single agent or technique has proven superior in preventing chronic post-ICU pain, multimodal strategies remain essential but underutilized in real-world burn care. Oral agents such as non-steroidal anti-inflammatory drugs and paracetamol may be adequate for minor burns but are insufficient for major injuries [23]. For extensive burns, opioids are often required in higher doses because of receptor alterations and neuroinflammatory changes [21]. Patient-controlled analgesia improves patient comfort but still requires careful monitoring due to altered pharmacokinetics during the hypermetabolic phase.

Experimental studies have demonstrated decreased opioid receptor density and increased expression of protein kinase C-γ and N-methyl-D-aspartate receptors following burn injury, which may contribute to tolerance and hyperalgesia and provide a rationale for the use of adjunctive agents such as ketamine [24].

Regional anesthesia represents a promising opioid-sparing strategy. Evidence from polytrauma populations has shown encouraging analgesic outcomes [25]. Peripheral nerve blocks interrupt nociceptive transmission and reduce primary and secondary sensitization, thereby limiting central “wind-up.” Ideally, regional techniques should be integrated within a broader multimodal analgesic plan. Both single-shot injections and continuous perineural infusion catheters are effective modalities for pain management in burn patients, each offering distinct advantages and limitations. In our clinical pathway, the need for prolonged analgesic coverage did not justify the placement of a continuous catheter, as donor sites are not manipulated and do not generate high NRS scores, and graft sites, although assessed daily, are not subjected to painful procedures.

There is no clear evidence that one technique is categorically superior for all burn patients; therefore, the choice should be individualized based on the clinical context [8,19,26,27]. We acknowledge that regional anesthesia may theoretically mask pain related to evolving compartment syndrome; therefore, in our institution nerve blocks are avoided in patients with clinical or surgical suspicion of compartment syndrome, and strict postoperative neurovascular monitoring is mandatory. Continuous perineural catheters extend analgesia for several days, facilitate bedside procedures such as dressing changes and debridement, and can reduce opioid requirements. The infection rate associated with perineural catheters in burn patients is low and comparable to that observed in other surgical populations, and major complications such as nerve injury are rare [26]. Continuous peripheral nerve blocks may temporarily reduce motor function, potentially limiting participation in early physiotherapy [28]. Several studies, including a trial comparing EMLA cream with the “3-in-1 block,” have demonstrated superior and longer-lasting donor-site analgesia with regional techniques in burn patients. A key limitation is that injections must be performed on intact skin, requiring careful planning and technique in this population. Current guidelines from the American Burn Association do not recommend one approach over the other because of limited high-quality evidence; both techniques should be considered as part of a multimodal strategy tailored to burn size, location, patient comorbidities, and institutional protocols.

Against this well-established background, the present retrospective study does not claim conceptual novelty regarding the benefits of regional anesthesia in burn care. Rather, it provides an incremental, protocol-based clinical contribution by describing a standardized and reproducible approach combining bilateral sciatic popliteal and adductor canal blocks for lower-limb escharectomy and autologous skin grafting. In our cohort, this combined regional strategy provided effective postoperative analgesia, minimized opioid requirements, and was not associated with block-related complications. These findings reinforce existing evidence supporting regional anesthesia as a safe and opioid-sparing modality in selected burn patients and suggest that standardized protocols may help reduce practice variability in real-world settings. In particular, our results are consistent with previous reports showing reduced postoperative pain scores and opioid requirements with peripheral nerve blocks in burn and trauma populations [25,26,27,28]. From a clinical perspective, these data suggest that standardized peripheral nerve block protocols may represent a feasible component of future opioid-sparing pathways in selected burn surgery patients.

In our institutional pathway, the first postoperative dressing change is routinely performed on postoperative day 5, when the skin graft is already adherent to the wound bed and does not require manipulation of pain-sensitive tissues. Subsequent dressing changes are performed on alternate days until complete wound healing is achieved. Similarly, donor-site dressings are managed conservatively, with only external dressing changes and no debridement or traumatic interventions. Under these conditions, wound care does not represent a clinically relevant painful stimulus, and this explains why pain during dressing changes was not included as a formal study endpoint.

Several limitations must be acknowledged. The monocentric, retrospective design may introduce selection and reporting bias. The small sample size limits generalizability and may not fully capture the heterogeneity of the broader burn population. The absence of a control group treated with general anesthesia or systemic analgesia alone prevents direct comparison between analgesic strategies. In addition, pain during wound care and dressing changes was not predefined as a formal endpoint, although it is reported descriptively in the text. Finally, follow-up beyond 30 days was not performed, leaving the long-term impact on chronic pain, functional recovery, and sustained opioid use unknown.

## 5. Conclusions

This retrospective study suggests that regional anesthesia may contribute to improved postoperative pain control in adult burn patients undergoing lower-limb escharectomy and autologous skin grafting. In our cohort, peripheral nerve blocks were associated with satisfactory analgesia and a limited need for rescue opioid administration, with no block-related complications observed.

These findings suggest that, in carefully selected patients, regional anesthesia may represent a safe adjunct within a multimodal analgesic strategy aimed at reducing systemic opioid exposure. However, this approach cannot be generalized to the broader burn population.

The conclusions of this study must be interpreted with caution because of the small sample size, retrospective design, and lack of long-term follow-up. The results should therefore be considered exploratory rather than definitive. Finally, from a practical clinical perspective, this study supports the feasibility of incorporating a standardized peripheral nerve block protocol into multimodal analgesic pathways for selected burn surgery patients. Future prospective, comparative, and adequately powered multicenter studies are needed to confirm these preliminary observations and to assess long-term outcomes.

## Figures and Tables

**Table 1:** Baseline characteristics of the study population (*n* = 25). TBSA: total body surface area.

Variable	Value
Age (years)	54 ± 12 (range 29–73)
Sex, *n* (%)	Male 15 (60%), Female 10 (40%)
TBSA (% of body surface area)	12 ± 4 (range 6–20)
Burn depth, *n* (%)	Deep dermal 12 (48%). Full-thickness 13 (52%)
Circumferential involvement, *n* (%)	Yes 9 (36%), No 16 (64%)
Anatomic region of the limb requiring surgery, *n* (%)	Thigh 7 (28%), Lower leg 13 (52%), Foot 5 (20%)
Comorbidities	Hypertension (32%), Diabetes mellitus 6 (24%), COPD 2 (8%), Obesity 1 (4%), Ischemic heart disease 1 (4%), None 7 (28%)

**Table 2:** Distribution of postoperative pain scores assessed using the Numerical Rating Scale (NRS) during the first 24 postoperative hours.

Pain Category	NRS Range	*n* (%)
No pain	0	8 (32%)
Mild pain	1–3	13 (52%)
Moderate pain	4–6	4 (16%)
Severe pain	7–10	0 (0%)

**Table 3:** 30-Day Readmission.

Readmission Within 30 Days	*n* (%)
Yes	0 (0%)
No	25 (100%)

## Data Availability

All data are available at University of Campania “L. Vanvitelli”.

## Abbreviations

The following abbreviations are used in this manuscript:

ICU	Intensive Care Unit
CPIP	Chronic Post-ICU Pain
BMI	body mass index
NRS	Numerical Rating Scale
NSAID	Non-steroidal anti-inflammatory drugs
CO_2_	Carbon dioxide
PCA	Patient controlled analgesia
PKC-γ	Protein kinase C gamma
NMDA	N-methyl-D-aspartate
